# Integrative genomic and functional characterization of a feline milk–derived *Lactiplantibacillus* plantarum MNN reveals host-specific adaptation and ecological safety

**DOI:** 10.3389/fmicb.2026.1742444

**Published:** 2026-01-21

**Authors:** Xinyu Gong, Xue Wang, Lu Chen, Huiming Huang, Min Wen

**Affiliations:** 1 Shandong Key Laboratory of Applied Technology for Protein and Peptide Drugs, Institute of Biopharmaceutical Research, Liaocheng University, Liaocheng, China; 2 Shandong Key Laboratory of Applied Technology for Protein and Peptide Drugs, School of Pharmaceutical Sciences and Food Engineering, Liaocheng University, Liaocheng, China; 3 Pet Nutrition Research and Development Center, Gambol Pet Group Co., Ltd., Liaocheng, China

**Keywords:** complete genome sequence, feline milk, *L. plantarum*, probiotic properties, ragdoll cat

## Abstract

Feline milk serves as a natural reservoir of host-adapted microorganisms that shape early-life gut microbiota and immune development. Our previous work identified *Pediococcus acidilactici* M22 from feline milk, which showed robust gastrointestinal tolerance, antioxidant capacity, and safety, providing the first evidence that feline milk–derived probiotics could be suitable for simulated pet milk formulations. However, *P. acidilactici* species possess limited genomic versatility and metabolic adaptability, warranting exploration of other lactic acid bacteria with broader functional repertoires. MNN exhibited superior acid and bile tolerance (99.83% survival at pH 2.5 and 88% at 0.3% bile) compared with M22 (59.93 and 84.38%, respectively), indicating enhanced gastrointestinal resilience. It demonstrated notable antioxidant capacity (DPPH 52.64%, ABTS 55.59%, superoxide 63.17%) and increased serum SOD and GSH while reducing MDA in mice, reflecting a stronger antioxidative defense than M22, whose effects were primarily systemic. Genome sequencing revealed a 3.29 Mb chromosome—1.23 Mb larger than M22—harboring 3,091 coding sequences enriched in stress response (*groEL, dnaK, trxA*), antioxidant (*katA, gshA*), and antimicrobial (*plnE*, *plnF*) genes, as well as expanded membrane transport and carbohydrate metabolism pathways. Unlike M22, MNN also preserved gut microbial homeostasis *in vivo*, maintaining *α*/*β* diversity and subtly enriching beneficial genera (*Oscillibacter, Adlercreutzia*) without dysbiosis. Functional prediction confirmed stable carbohydrate and amino acid metabolism, with no enrichment of resistance or virulence genes. Compared with *P. acidilactici* M22, *L. plantarum* MNN exhibits higher genomic plasticity, stronger antioxidative capacity, and distinct ecological compatibility, marking a functional transition from “safety-verified probiotic” to “host-adapted microbiota-stabilizing probiotic.” Integrating genomic, functional, and ecological analyses, this study identifies MNN as a next-generation probiotic candidate for enhancing intestinal homeostasis and antioxidant defense in companion animals.

## 1 Introduction

According to the accepted definition, probiotics are viable microorganisms capable of promoting host health when consumed in adequate amounts, largely by influencing gut microbial balance (Zhao et al., 2024). Numerous studies have established that the probiotics play a pivotal role in various aspects of animal health, including metabolic regulation and energy homeostasis, immune stimulation, maintenance of intestinal barrier integrity, and modulation of neurobehavioral functions (Hill et al., 2014). Indeed, the addition of probiotics to pet diets is considered a beneficial practice that contributes to improving the overall health status of companion animals. This approach offers multiple advantages, including enhanced gut health, immune modulation, and the potential to serve as an alternative to antibiotics in pet healthcare (Hou et al., 2021).

Breast milk is regarded as the optimal food for neonates due to its containing a well-balanced all nutrients required for growth and protective molecules such as immunoglobulins, lactoferrin, and lysozyme (Hanson et al., 2003). In addition, recent findings have revealed that breast milk contains a substantial population of beneficial bacteria and is regarded as the critical source for gut microbiota (Brockway, 2024; Li et al., 2024). Indeed, probiotics such as *L. plantarum* isolated from giant panda milk, *Bifidobacteria* isolated from human milk, and *Pediococcus acidilactici* isolated from canine milk have all demonstrated beneficial properties through extensive characterization and screening (Damaceno et al., 2023; Liu et al., 2024; Liu et al., 2025). Compared to feces-derived probiotics, breast milk-derived probiotics exhibit several advantages, including natural origin, enhanced safety, and improved immunological compatibility with the host (Obisesan et al., 2024). Despite its potential, feline milk has rarely been investigated as a source of probiotic strains, indicating that more research is warranted in this area.

*L.plantarum* is a widely adaptable and functionally versatile lactic acid bacteria (LAB) species demonstrating substantial promise in promoting gastrointestinal health across diverse animal hosts (Garcia-Gonzalez et al., 2021). The combination of acid and bile tolerance, antioxidant potential, immunomodulatory functions, and activity against diverse pathogens supports its designation as a strong probiotic candidate (Singhal et al., 2021; Wu et al., 2022; Zhang et al., 2023; Lee et al., 2024). Feline, as obligate carnivores, possess a relatively short and simple GI tract, resulting in a uniquely specialized microbiota (Rochus et al., 2014). This microbiota might harbor *L. plantarum* with distinct beneficial characteristics. Earlier investigations revealed that *L. plantarum* isolated from feline feces possess strong acid tolerance, notable antioxidant activity, high cell-surface hydrophobicity, and excellent adherence capacity (Liang et al., 2024). Nevertheless, systematic studies on *L. plantarum* isolated specifically from feline milk remain sparse, particularly concerning strain screening, comprehensive functional characterization, and assessment of probiotic potential.

As probiotics gain broader application, safety considerations have concurrently become crucial. Potential risks, such as the transfer of antibiotic resistance genes and opportunistic pathogenicity, necessitate rigorous safety evaluations at the strain-specific level (Pradhan et al., 2020). Whole-genome sequencing (WGS) technology serves as a robust approach to uncover detailed genetic information, including virulence determinants, antibiotic resistance genes, and beneficial functional genes (Merenstein et al., 2023). This genomic-level insight allows a deeper understanding of the mechanisms underlying probiotic characteristics and provides a reliable basis for evaluating probiotic safety and efficacy (Syrokou et al., 2022; Deng et al., 2023).

Therefore, the current study aimed to isolate *L. plantarum* from feline milk and systematically evaluate their traits *in vitro* probiotic and biosafety profile. A representative L. Plantarum strain exhibiting strong probiotic potential was subsequently selected for WGS analysis to explore its genomic characteristics and functional capacities. This research provides essential theoretical support and scientific foundations for the potential application of feline milk-derived *L. plantarum* in pet food formulations and gastrointestinal health interventions.

## 2 Materials and methods

### 2.1 Sample collection

Two randomly selected Ragdoll cats confirmed to be healthy served as the sample donors (Zhao et al., 2024). Ethical approval for feline milk collection was obtained from the Institutional Animal Care and Use Committee (IACUC) of Liaocheng University (AP2024022959). None of the animals had been administered antibiotics or probiotics during the 90 days preceding sampling. Samples were collected under aseptic conditions.

### 2.2 Obtaining of MNN

Milk samples were spread onto MRS agar plates (Haibo, China) and incubated at 37 °C for 24 h. Amplified products were sequenced by Shanghai Majorbio Bio-pharm Technology Co., Ltd. (Shanghai, China), and phylogenetic relationships were inferred using MEGA version 7.0.(Kumar et al., 2016). Species identification of strain MNN was performed based on 16S rRNA gene sequencing and further confirmed by whole-genome-based analyses, including average nucleotide identity (ANI) and phylogenomic analysis. Given the higher taxonomic resolution provided by whole-genome sequencing, additional single-gene markers such as rpoB or pheS were not separately employed.

The *L. plantarum* MNN, previously isolated and taxonomically characterized in our laboratory, was employed in this study. It has been deposited in the China Center for Type Culture Collection (CCTCC NO.31003).

### 2.3 *In vitro* characterization of probiotic traits

#### 2.3.1 Growth kinetics and acid production

For growth and acid production assays and samples were withdrawn every 2 h to assess OD and pH. Bacterial growth was monitored by measuring optical density at 600 nm (OD600), which was used as a rapid and widely accepted indicator to determine growth dynamics and define different growth phases. Growth curve experiments were performed in biological triplicates, and the averaged values are presented. Although CFU enumeration can provide more precise viable cell counts, OD600 measurement was considered sufficient for identifying growth phases required for subsequent experiments in this study.

#### 2.3.2 Gastrointestinal tolerance assay

Gastrointestinal tolerance was assessed according to the method reported by Safi et al.(Safi et al., 2023). For evaluating gastric acid resistance, sterile MRS broth was adjusted to pH values of 2.5, 3.0, and 4.0, with the pH reconfirmed post-sterilization. Subsequently, 100 μL of overnight bacterial culture was inoculated into 4.9 mL of acidified MRS broth and incubated anaerobically. Samples were collected at 0 and 3 h and spread onto MRS. After 24 h of incubation, colonies were counted to determine viable cell numbers.

The bile salt tolerance assay was performed following the same procedure as described above for acid resistance.

### 2.4 Evaluation of antioxidant properties

Antioxidant assays were performed using bacterial cell-free extracts prepared by ultrasonic disruption followed by centrifugation to remove cell debris. The ability to scavenge DPPH radicals was assessed using a commercial assay kit (Nanjing Jiancheng Bioengineering Institute, China). Superoxide anion scavenging capacity was assessed with a commercial assay kit (Solarbio, Beijing, China) according to the manufacturer’s instructions. Subsequently, 0.02 mL of the sample was added to 0.2 mL of the working solution, incubated for 30 min at 20–25 °C, and the absorbance at 734 nm was recorded to evaluate scavenging activity (Zhao et al., 2024).

Autoaggregation Activity.

Stationary-phase bacterial cultures were rinsed three times with PBS (pH 7.0), and the OD600 value (A0)of the resulting suspension was recorded. After vortexing for 25–30 s, the suspension was incubated at 37 °C for 8 h. The OD600 (A1) was measured using a spectrophotometer. Calculated using Equation 1.


(1) Autoaggregation activity(AAG,%)=(1−A1/A0)×100%

### 2.5 Cell surface hydrophobicity

MNN was grown at 37 °C for 18–20 h and subjected to centrifugation at 8,000 × g for 5 min. Following three washes with PBS (pH 7.0), the cells were resuspended to an OD₆₀₀ of 0.25 ± 0.05 (A₂). Followed by vortexing for 120 s and incubation at 37 °C for 3 h. The OD600 of the aqueous phase was then measured (A3). Calculated using Equation 2.


(2) Cell Surface Hydrophobicity(CSH,%)=(1−A3/A2)×100%

### 2.6 Assessment of bacterial adhesion to Caco-2 cells

#### 2.6.1 FITC-based fluorescent labeling of probiotic strains

The labeling procedure was carried out as outlined by Kaktcham et al., with modifications to suit this study (Kaktcham et al., 2018).

#### 2.6.2 Adhesion assay

Caco-2 cells between passages 20–40 were used for adhesion assays. Monolayer integrity was assessed by visual confluence prior to experiments. Fluorescence intensity was used as a relative indicator of bacterial adhesion and was normalized to the initial fluorescence signal to calculate adhesion rates (%). The adhesion capacity of the bacterial strains to intestinal epithelial cells was evaluated using a Caco-2 cell monolayer model. Briefly, Caco-2 cells were seeded into 12-well culture plates at a density of 5 × 10^5^ cells/mL and incubated at 37 °C in a humidified atmosphere containing 5% CO₂ for 24 h to allow monolayer formation. After incubation with the fluorescently labeled bacteria, the wells were gently washed three times with phosphate-buffered saline (PBS) to remove non-adherent bacteria. Subsequently, the Caco-2 cells were detached using 0.25% trypsin, and the fluorescence intensity of the cell suspensions was measured using a microplate reader at an excitation wavelength of 485 nm and an emission wavelength of 530 nm. The adhesion ability was expressed as fluorescence intensity, reflecting the amount of bacteria adhered to the Caco-2 cells.(Kaktcham et al., 2018).

### 2.7 Evaluation of safety-related properties of the isolated strains

#### 2.7.1 Hemolytic activity

Hemolytic activity was tested on Columbia blood agar (Nataraj et al., 2023). *Staphylococcus aureus* was included as a positive control to verify hemolytic activity.

#### 2.7.2 Assessment of antibiotic susceptibility

One hundred microliters of bacterial suspension was spread onto pre-prepared MRS agar plates. Between five and six antibiotic disks were positioned on each agar plate and left at 4 °C for 12 h to permit pre-diffusion (Nataraj et al., 2023).

### 2.8 Evaluation of antimicrobial potential

MNN was grown in MRS broth at 37 °C, after which the culture was centrifuged to obtain the cell-free supernatant and resuspended to 1 × 10^9^ CFU/mL. Pathogenic strains (*E. coli* O157, *S. typhimurium* SL1344, and *S. aureus*) adjusted to 1 × 10^7^ CFU/mL. Antimicrobial activity was tested by the Oxford cup method: 200 μL of bacterial suspension, supernatant, pellet, or PBS (control) was added to wells on LB agar, incubated for 24 h at 37 °C, and inhibition zone diameters were recorded.

### 2.9 *In vivo* toxicological and safety evaluation of MNN in mice

#### 2.9.1 Ethics statement

Animal care and procedures were carried out in accordance with the Laboratory Animal Ethics Committee of Liaocheng University (Liaocheng, China; approval no. AP2024022959) and with the National Institutes of Health Laboratory Animal Care and Use Guidelines (NIH Publication No. 80–23).

#### 2.9.2 Animal experiments

Forty six-week-old ICR mice (20 males and 20 females) were purchased from Nanjing Huimiaoxin (China). After a 7-day acclimation at 25 °C with free access to food and water, the animals were randomly allocated into four groups (n = 10). For 28 days, the control group (C) was given 200 μL of sterile saline by gavage, while the low- (L:1 × 10^7^ CFU/mL), medium- (M:1 × 10^8^ CFU/mL), and high-dose (H:1 × 10^9^ CFU/mL) groups received 0.2 mL of *L. plantarum* MNN. The gavage volume was standardized and adjusted according to mouse body weight to ensure consistent dosing. Clinical signs, including diarrhea, were monitored daily, and body weights were recorded weekly. At the end of the experiment, mice were fasted for 12 h before blood collection from the orbital sinus. After euthanasia by cervical dislocation, gross pathology was examined, major organs were excised and weighed (Li et al., 2019).

#### 2.9.3 Evaluation of serum biochemical indices in mice

Serum biochemical parameters, including hepatic and renal function indices as well as antioxidant markers, were determined using commercial kits (Jiancheng Bioengineering Institute, China).

### 2.10 Gut microbiota diversity and composition analysis

For gut microbiota analysis, cecal contents were collected under sterile conditions immediately after euthanasia at the end of the experimental period, following the final administration. The collected samples were snap-frozen in liquid nitrogen and stored at −80 °C until DNA extraction for 16S rRNA gene sequencing.

#### 2.10.1 DNA extraction and PCR amplification

Total microbial genomic DNA was isolated from the samples with the E. Z. N. A.® soil DNA Kit (Omega Bio-tek, Norcross, GA, U. S.) following the manufacturer’s protocol (Wang et al., 2007). DNA purity and concentration were assessed by 1.0% agarose gel electrophoresis and a NanoDrop2000 spectrophotometer (Thermo Scientific, United States), and samples were stored at −80 °C until further analysis (Liu et al., 2016). The V3–V4 hypervariable region of the bacterial 16S rRNA gene was amplified employing primer pairs 338F (5’-ACTCCTACGGGAGGCAGCAG-3′) and 806R (5’-GGACTACHVGGGTWTCTAAT-3′) using a T100 Thermal Cycler (BIO-RAD, USA). Standard PCR amplification was performed using specific primers and a commercial master mix. The resulting products were then visualized via electrophoresis, purified, and quantified fluorometrically for downstream applications (Chen et al., 2018).

#### 2.10.2 Data processing

Raw FASTQ data underwent demultiplexing, quality filtering, and assembly using custom scripts and bioinformatics tools. Quality-controlled sequences were then clustered into OTUs at 97% similarity, followed by removal of chloroplast-derived sequences. To mitigate sequencing depth bias, all samples were rarefied to 20,000 sequences per sample (Chen et al., 2018).

Taxonomic assignment of OTUs was performed using the RDP Classifier (v11.5) against the Silva v138.2 database with a confidence threshold of 0.7. Metagenomic functions were predicted with PICRUSt2, which placed representative sequences into a reference tree using EPA-NG and Gappa, normalized copy numbers with Castor, and predicted gene pathways via MinPath, following the recommended workflow (Magoč and Salzberg, 2011).

### 2.11 Whole-genome sequencing (WGS)

#### 2.11.1 Extraction of genomic DNA

L.plantarum MNN was streaked onto de MRS agar and maintained at 37 °C for 24 h. A single colony was then inoculated into 0.2 L of MRS broth and cultured at 37 °C for approximately 16 h with shaking at 200 rpm. The bacterial cells were harvested by centrifugation at 8000 × g for 5 min.

#### 2.11.2 Sequencing libraries and whole-genome sequencing

Whole-genome sequencing was performed using a hybrid approach combining the PacBio Sequel IIe and Illumina NovaSeq 6,000 platforms. For Illumina sequencing, genomic DNA was fragmented into 400–500 bp fragments with a Covaris MNN Focused Acoustic Shearer, and libraries were prepared using the NEXTFLEX Rapid DNA-Seq Kit (PerkinElmer, USA). Library construction involved end repair and phosphorylation of 5′ ends, A-tailing of 3′ ends, adapter ligation, and PCR enrichment.

#### 2.11.3 Genome assembly

All analyses were conducted using the tools provided by the Majorbio Cloud Platform (http://cloud.majorbio.com), operated by Shanghai Majorbio Bio-pharm Technology Co., Ltd. (Shanghai, China). The high-fidelity (HiFi) reads generated from the PacBio platform were subsequently used for downstream analysis. Cleaned Illumina reads and PacBio HiFi reads were co-assembled using Unicycler (v0.4.8) to generate complete genome sequences (Wick et al., 2017) and Pilon (v1.22) was employed to polish the genome assembly using short-read alignments, thereby reducing the rate of small errors. The final assembled genome was submitted to the NCBI database (accession number PRJNA1285844). The coding sequences (CDs) of chromosome and plasmid were predicted using Glimmer or Prodigal v2.6.3(Hyatt et al., 2010) and GeneMarkS (Besemer and Borodovsky, 2005) respectively. tRNA-scan-SE (v 2.0)(Chan and Lowe, 2019) was used for tRNA prediction and Barrnap v0.9 (https://github.com/tseemann/barrnap) was used for rRNA prediction. The predicted coding sequences (CDSs) were annotated using multiple databases, including NR, Swiss-Prot, Pfam, GO, COG, KEGG, and CAZy, through sequence alignment tools such as BLAST, DIAMOND, and HMMER. Briefly, each set of query proteins was aligned against the respective databases, and the annotations of the best-matching hits (e-value < 1e−5) were retrieved for functional assignment.

### 2.12 Statistical analysis

Data are presented as mean ± standard deviation (SD). Statistical analyses were conducted with GraphPad Prism 9.0 (GraphPad Software, San Diego, CA, USA). Differences between two groups were assessed using unpaired Student’s t-tests, and multiple-group comparisons were evaluated by one-way analysis of variance (ANOVA). One-way ANOVA was followed by Tukey’s multiple comparison test where appropriate. A *p*-value < 0.05 was considered statistically significant.

## 3 Results

### 3.1 Isolation of MNN

**Figure 1:**
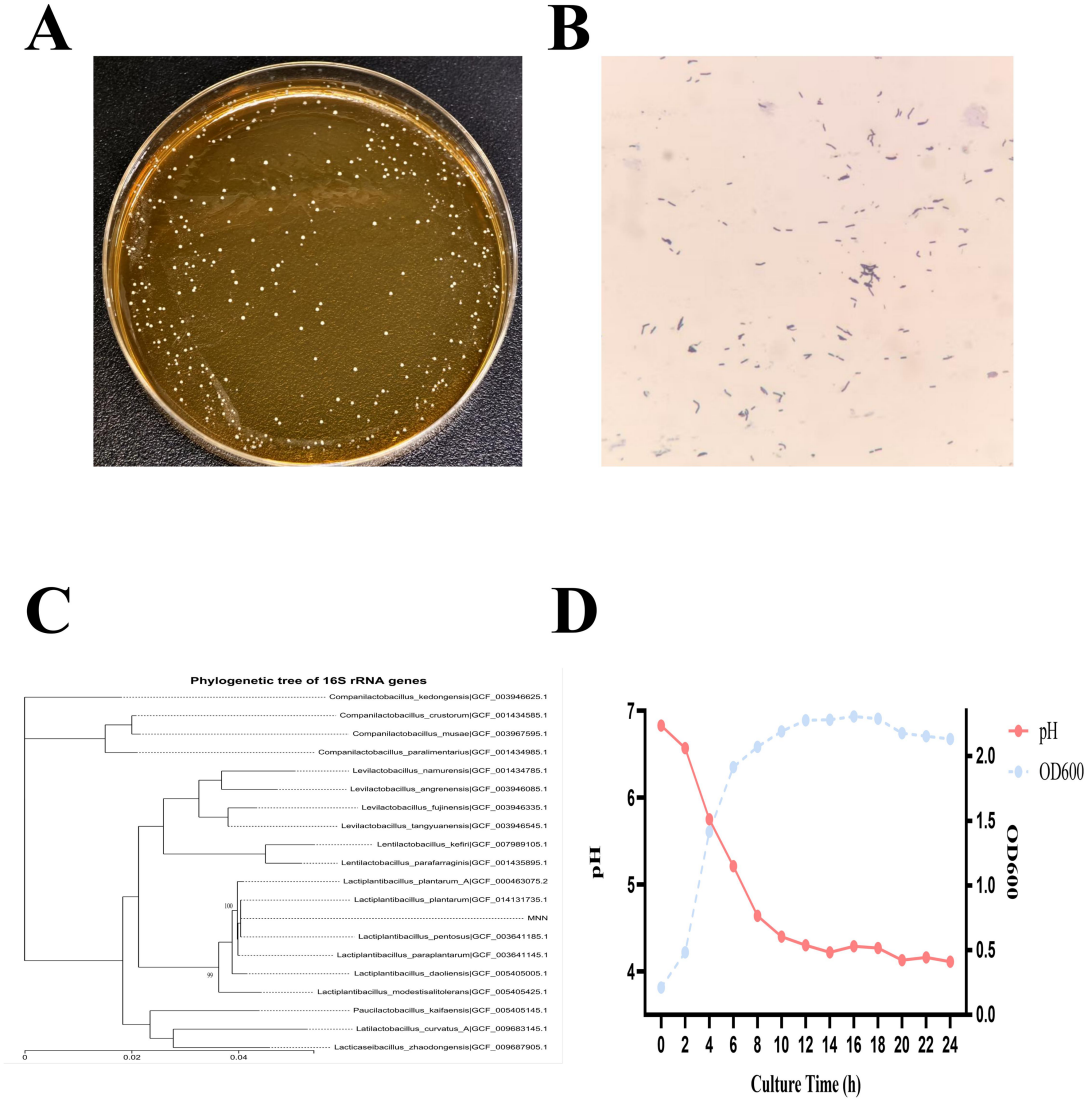
Morphology, staining, phylogenetic analysis, and growth characteristics of strain MNN. **(A)** Colony morphology of strain MNN on MRS agar. **(B)** Gram staining results of strain MNN. **(C)** Neighbor-joining phylogenetic tree of strain MNN based on 16S rRNA gene sequences. **(D)** Growth curve and pH changes of strain MNN in MRS broth over a 24 h incubation period.

MNN was collected from the milk of a healthy cat. *L. plantarum* MNN produced circular, opaque, creamy-white colonies with smooth, convex surfaces and entire margins, consistent with the typical morphology of this species (Figure 1A). Gram staining showed Gram-positive, non-spore-forming rods, occurring singly, in pairs, or occasionally in short chains (Figure 1B). The 16S rRNA gene of MNN was amplified and sequenced, and BLAST analysis against the GenBank database revealed 100% identity to *L. plantarum*, confirming its taxonomic assignment (Figure 1C).

### 3.2 Screening of properties

#### 3.2.1 Characterization of growth and acidification dynamics of MNN

MNN exhibited a typical bacterial growth pattern, remaining in the lag phase during the initial 0–2 h, entering the exponential (log) growth stage after 2 h, and reaching the Late growth phase at approximately 12 h. MNN was characterized by a notable capacity for acid production.(Figure 1D).

#### 3.2.2 Gastrointestinal tolerance assay

**Figure 2:**
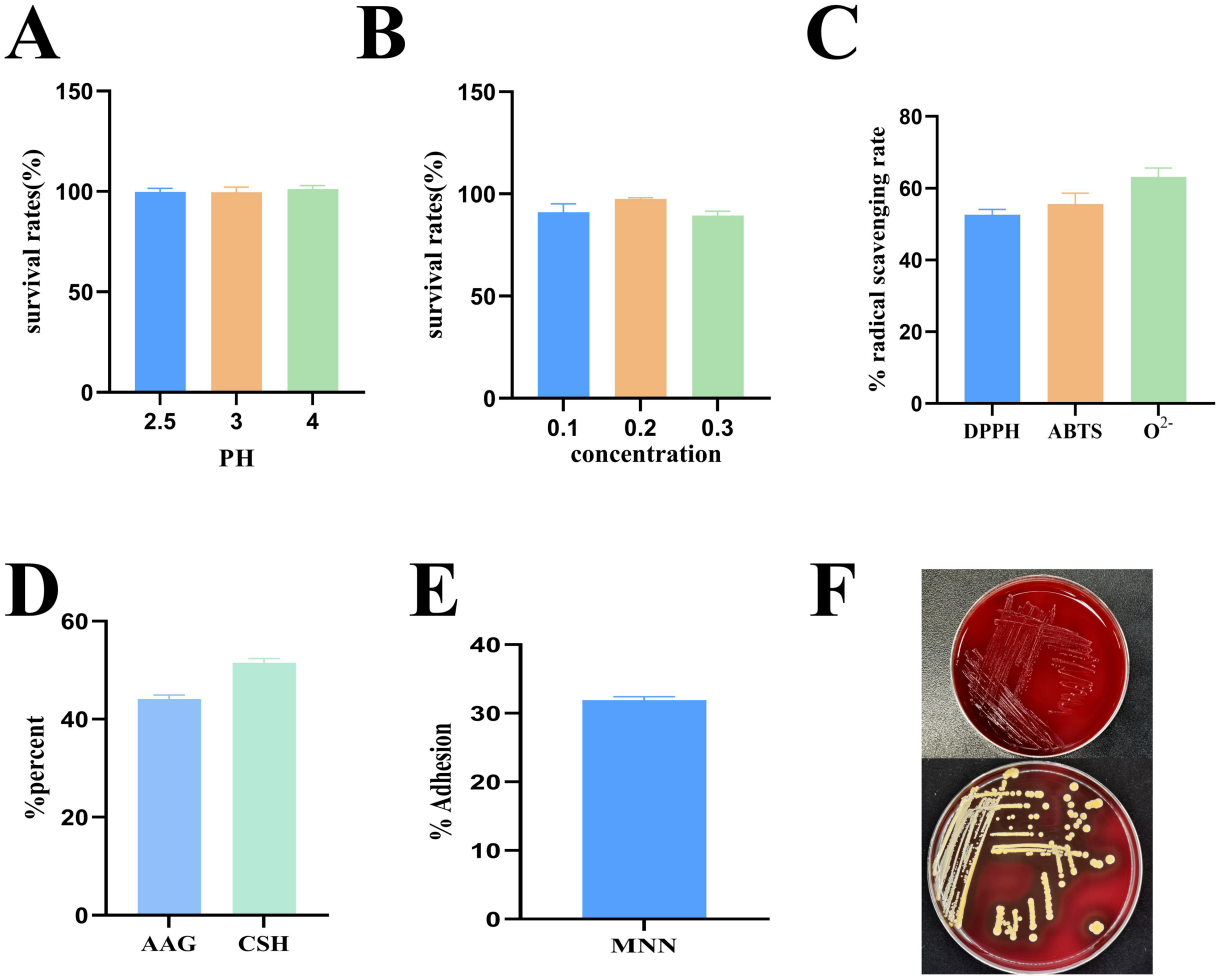
*In vitro* evaluation of probiotic potential. **(A)** pH 2.5–4 acid resistance; **(B)** 0.1–0.3% bile salt resistance; **(C)** Radical scavenging rate; **(D)** Autoaggregation ability (AAG) and cell surface hydrophobicity (CSH); **(E)** Adhesion to Caco-2 cells; **(F)** Hemolysis tests.

As shown in Figures 2A,B, MNN exhibited a survival percentage of 99.83% at pH = 2.5 and 88.00% under 0.3% bile salt, demonstrating high resistance to both acidic and bile stress. Low concentrations of bile salts may act as environmental signals that induce stress adaptation mechanisms, such as bile salt hydrolase activity and membrane remodeling, thereby promoting bacterial growth in bile-tolerant strains. These results highlight its robust gastrointestinal tolerance and support its potential as a promising probiotic candidate.

#### 3.2.3 Assessment of antioxidant capacity

As shown in Figure 2C, MNN demonstrated considerable antioxidant capacity, with scavenging activities of 52.64% for DPPH radicals, 55.59% for ABTS radicals, and 63.17% for superoxide anions.

### 3.3 Cell adhesion ability of MNN

MNN exhibited 44.12% auto-aggregation and 51.48% cell surface hydrophobicity (Figure 2D). In addition, it achieving an adhesion rate of 31.90% (Figure 2E).

### 3.4 Safety characteristics of MNN

#### 3.4.1 Hemolytic activity

As shown in Figure 2F, MNN exhibited no hemolytic activity (*γ*-hemolysis), indicating that the strain can be regarded as safe.

#### 3.4.2 Antimicrobial resistance characteristics of MNN

As shown in Table 1, strain MNN exhibited sensitivity to several antibiotics, including gentamicin, erythromycin, ampicillin, penicillin, cefazolin, tetracycline, and chloramphenicol. In contrast, it was resistant to dibekacin, vancomycin, norfloxacin, ciprofloxacin, and sulfamethoxazole–trimethoprim.

**Table 1:** Antibiotic resistance of strain MNN.

Antibiotic class	Antibiotic	Inhibition zone diameter (mm)	Susceptibility (S/I/R)
Aminoglycosides	Gentamicin	24 ± 0.13	S
	Dibekacin	0	R
Glycopeptides	Vancomycin	0	R
Macrolides	Erythromycin	44 ± 0.22	S
β-Lactams	Ampicillin	52 ± 0.25	S
	Penicillin	52 ± 0.24	S
	Cefazolin	48 ± 0.20	S
Tetracyclines	Tetracycline	30 ± 0.15	S
Fluoroquinolones	Norfloxacin	0	R
	Ciprofloxacin	0	R
Sulfonamides / Antimetabolites	Sulfamethoxazole-trimethoprim	0	R
Phenicols	Chloramphenicol	50 ± 0.11	S

### 3.5 Antimicrobial effects of MNN

**Figure 3:**
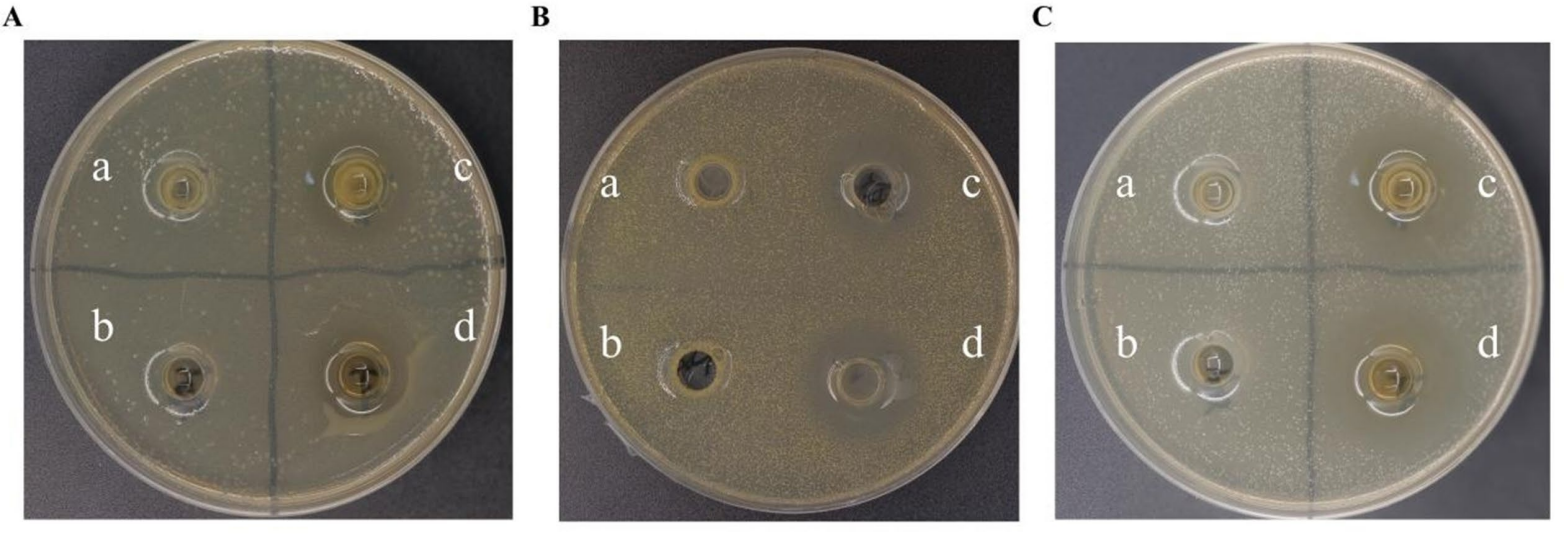
The inhibitory effects of *L. plantarum* MNN against pathogenic bacteria. **(A)** *Escherichia coli* O157; **(B)** *Staphylococcus aureus*; **(C)** *Salmonella Typhimurium* SL1344. On each LB plate, the following treatments were applied: **(A)** PBS (blank control); **(B)** Bacterial pellet of MNN; **(C)** Bacterial suspension of MNN; **(D)** Cell-free supernatant of MNN.

No inhibitory effects were observed for either the bacterial protein fraction or PBS (negative control) (Figures 3A,B). The bacterial suspension and its supernatant produced similar inhibition zones toward the tested pathogens (Figures 3C,D). These findings demonstrate that *L. plantarum* MNN possesses strong inhibitory activity against pathogenic bacteria. Quantitative data for inhibition zone diameters are provided in Table 2. All assays were performed in triplicate (n = 3), and statistical significance was assessed by one-way ANOVA.

**Table 2:** Inhibition zone diameters (mm) of *L. plantarum* MNN against pathogenic bacteria (mean ± SD, *n* = 3).

Pathogenic bacteria	PBS (blank control) (mm)	Bacterial pellet (mm)	Bacterial suspension (mm)	Cell-free supernatant (mm)
*Escherichia coli O157*	0	0	20.00 ± 1.00**	22 ± 2.00**
*Staphylococcus aureus*	0	0	22.00 ± 2.00**	24.00 ± 1.80**
*Salmonella Typhimurium SL1344*	0	0	24 ± 2.00**	26.00 ± 1.00**

“0” indicates no inhibition zone. Statistical significance compared with PBS control: **p < 0.01.

### 3.6 Safety evaluation *in vivo*

**Figure 4:**
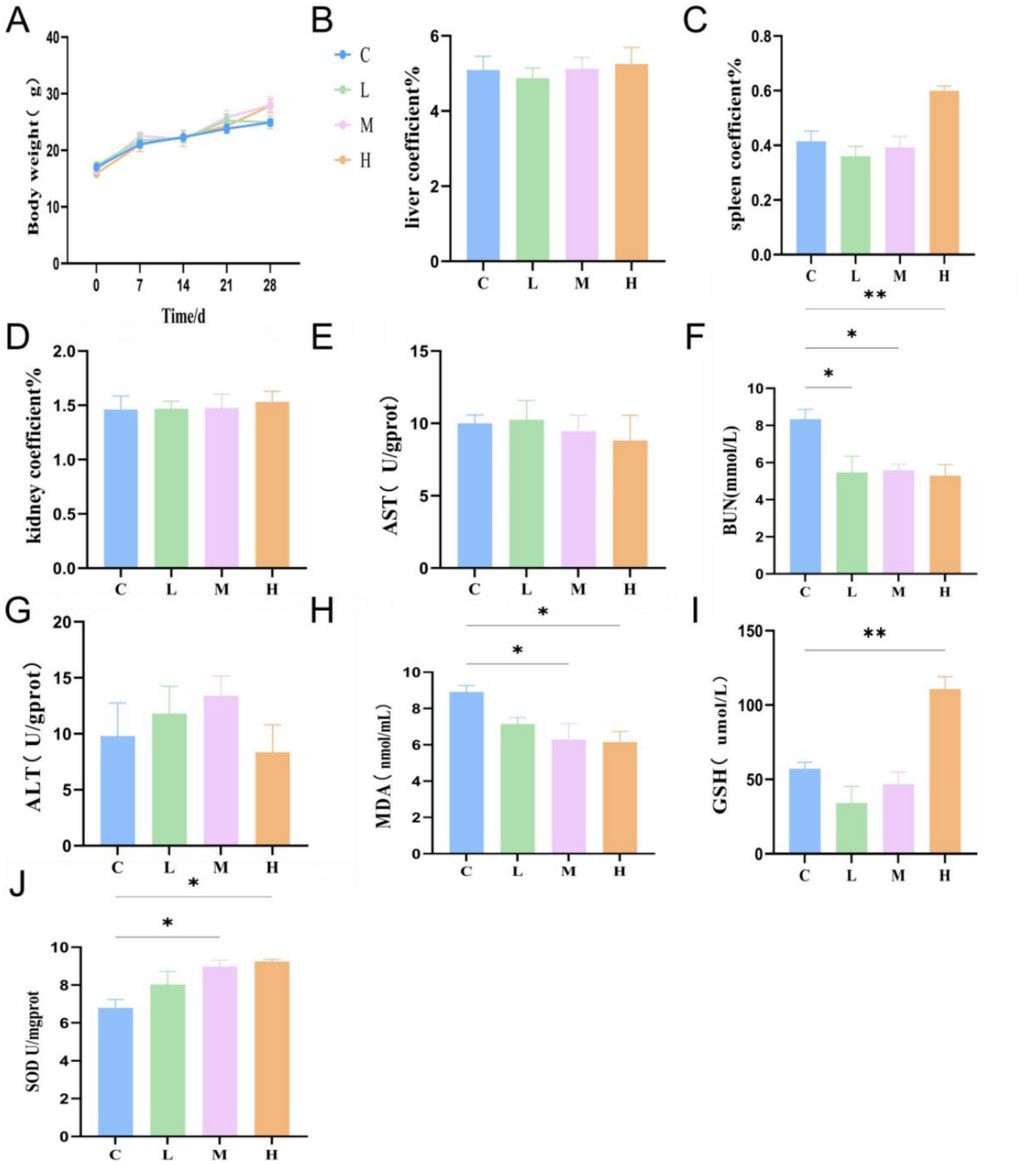
MNN safe evaluation *in vivo*. **(A)** Body weight; **(B)** Liver coefficient; **(C)** Spleen coefficient; **(D)** Kidney coefficient; **(E)** Aspartate amino transferase; **(F)** Blood urea nitrogen; **(G)** Alanine aminotransferase; **(H)** Malondialdehyde; **(I)** Glutathione in mice; and **(J)** Superoxide dismutase. *p < 0.05, **p < 0.01 and indicate differences between different types of treatment of *L. plantarum* MNN, *n* = 10.

*In vivo* safety was examined using a 28-day oral administration model in mice, where *L. plantarum* MNN was provided at low (L), medium (M), and high (H) doses, with sterile saline serving as the control (C). Throughout the experimental period, no significant differences in body weight gain were observed among all groups (Figure 4A), indicating that MNN administration did not adversely affect growth or general health status. Organ coefficients for the liver, spleen, and kidney remained statistically unchanged across all groups (Figures 4B–D), suggesting no detectable organ swelling or atrophy associated with MNN intake. To assess hepatic and renal function, serum biochemical markers were analyzed. There were no significant changes in AST (Figure 4E) and ALT (Figure 4G) levels among all groups, indicating that MNN did not induce hepatic injury. Notably, Blood Urea Nitrogen (BUN) levels were significantly reduced in M, L, and H groups compared to the control (Figures 4F, *P* < 0.05, *p* < 0.01), suggesting improved renal metabolic function or reduced nitrogenous waste accumulation following probiotic administration. Markers of oxidative stress were also examined. MDA levels, a marker of lipid peroxidation, were significantly lower in the L and H groups compared to the control (Figures 4H, *P* < 0.05), indicating reduced oxidative damage. Additionally, GSH levels were elevated in the H group (Figure 4I), while SOD activity was significantly increased in the M and H groups compared to the control (Figures 4J*P* < 0.05). These findings suggest that MNN enhances the host’s antioxidant capacity in a dose-dependent manner. Collectively, these results demonstrate that oral administration of *L. plantarum* MNN at various doses is safe in mice, with no observed toxicity or organ dysfunction, and may confer antioxidant benefits through modulation of oxidative stress markers.

### 3.7 Gut microbiota diversity

#### 3.7.1 *α*-diversity

**Figure 5:**
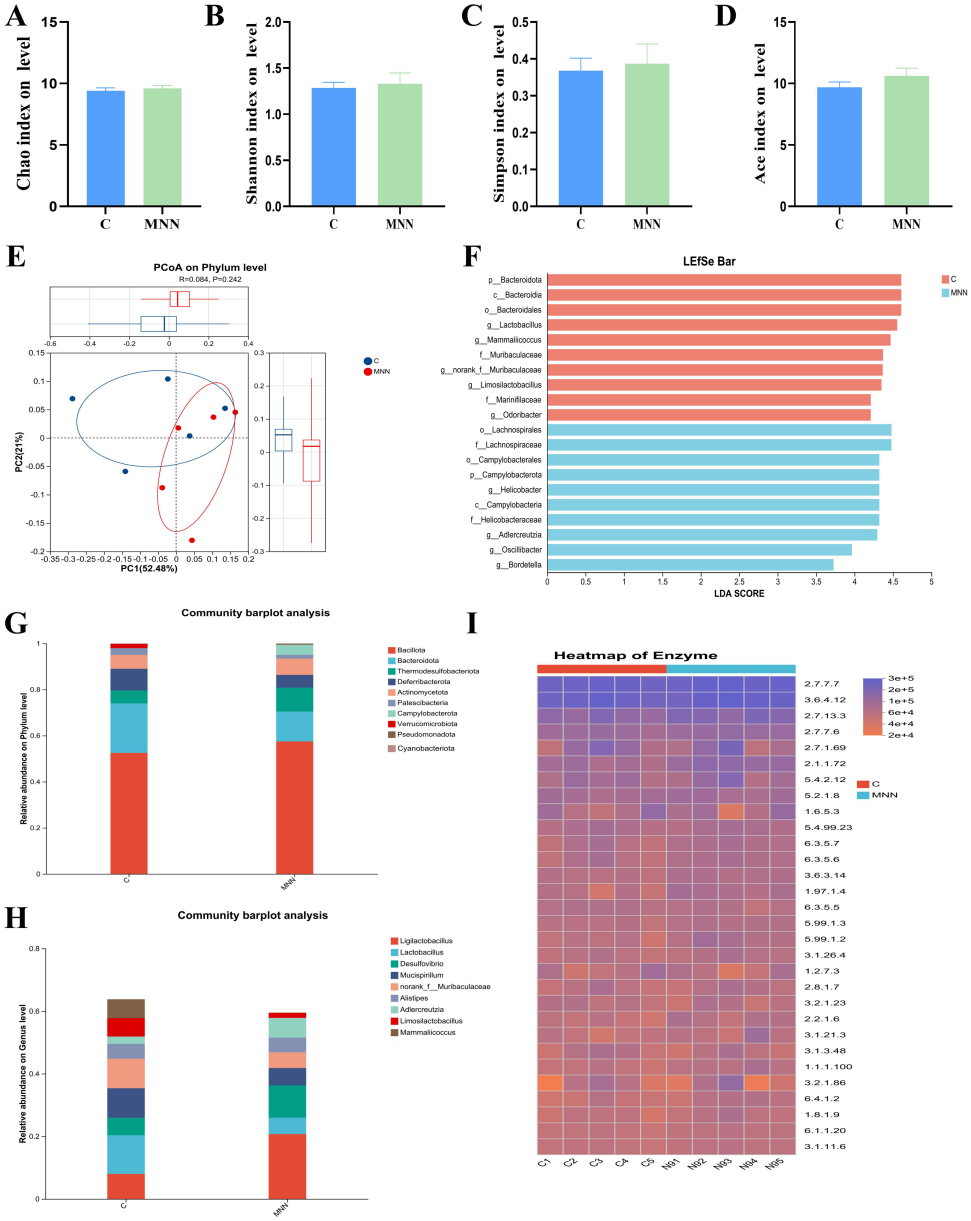
Gut microbiota diversity. Comparison of α-diversity indices **(A)** Chao1, **(B)** Shannon, **(C)** Simpson, **(D)** ACE between the C and MNN groups at the phylum level. **(E)** Principal coordinate analysis (PCoA) based on phylum-level community composition in the control (C, blue) and MNN (red) groups. **(F)** Linear discriminant analysis effect size (LEfSe) identifying differential taxa between the C (red) and MNN (blue) groups. **(G)** and **(H)** Relative abundance of gut microbiota at the phylum (bottom) and genus (top) levels in the C and MNN groups. **(I)** Heatmap of predicted enzyme abundances (EC numbers) in the gut microbiota of C and MNN groups, as inferred by PICRUSt2.

To assess the impact of *L. plantarum* MNN administration on the richness and diversity of the intestinal microbiota, multiple α-diversity indices were calculated at the phylum level. As shown in Figures 5A–D, the Shannon and Simpson indices exhibited no significant differences between the C and MNN groups, indicating comparable community diversity and evenness. The Chao1 index also remained similar between the two groups, suggesting that microbial richness was not altered by probiotic treatment. Although the ACE index of the MNN group appeared slightly lower than that of the control group, the difference did not reach statistical significance due to large inter-individual variability.

#### 3.7.2 *β*-diversity

To further determine whether oral administration of *L. plantarum* MNN influenced the overall microbial community structure, β-diversity was evaluated using principal coordinate analysis (PCoA) at the phylum level. As shown in Figure 5E, samples from the control (C) and MNN groups were largely intermixed, without clear clustering or separation. The first two principal coordinates (PC1 and PC2) explained 52.48 and 21.0% of the total variation, respectively. Statistical analysis by ANOSIM revealed a low R value (R = 0.084) and a non-significant difference between groups (*p* = 0.242), suggesting that community structures were comparable across treatments.

#### 3.7.3 Microbial composition

The relative abundance of gut microbiota was further compared at the phylum and genus levels (Figures 5G,H). At the phylum level, both groups were dominated by Bacillota and Bacteroidota, followed by Thermodesulfobacteriota and Deferribacterota, with no major compositional differences observed between the C and MNN groups. The overall Bacillota/Bacteroidota (F/B) ratio remained comparable across treatments, suggesting that MNN administration did not disrupt the dominant phylum-level structure of the gut microbiota.

At the genus level, the predominant taxa included Ligilactobacillus, Lactobacillus, Desulfovibrio, Alistipes, and members of the Muribaculaceae family. Although minor fluctuations in relative abundance were observed (e.g., a slightly higher proportion of Ligilactobacillus in the MNN group), no obvious enrichment of potentially pathogenic genera (such as Escherichia-Shigella or Enterococcus) was detected. The overall genus-level composition remained stable, indicating that MNN supplementation did not cause dysbiosis or promote the proliferation of opportunistic pathogens.

Collectively, these results demonstrate that oral administration of MNN preserved the major taxonomic structure of the gut microbiota at both phylum and genus levels, further confirming its *in vivo* safety.

#### 3.7.4 Differential taxa

To further identify taxa that contributed to group differences, linear discriminant analysis effect size (LEfSe) was conducted. As shown in Figure 5F, several taxa were enriched in the control group, including Bacteroidota (phylum), Bacteroidia (class), Bacteroidales (order), and genera such as Lactobacillus, Mammaliicoccus, and members of the Muribaculaceae family. In contrast, the MNN group was characterized by the enrichment of taxa such as Campylobacterota (phylum), Campylobacteria (class), Campylobacterales (order), Helicobacter, Adlercreutzia, Oscillibacter, and Bordetella.

Although certain differential genera were identified between groups, most belonged to commensal or low-abundance taxa. Importantly, no significant enrichment of pathogenic bacteria (e.g., Escherichia-Shigella or Enterococcus) was observed.

#### 3.7.5 Functional prediction

To explore the potential functional impact of *L. plantarum* MNN supplementation on the gut microbiota, PICRUSt2 was employed to predict microbial functional profiles at the enzyme (EC number) level. As shown in Figure 5I, the overall distribution of predicted enzyme abundances was broadly similar between the control (C) and MNN groups, with no distinct clustering or group-specific enrichment patterns. Most enzymes associated with basic metabolic pathways, including carbohydrate metabolism (e.g., EC 3.2.1.23, EC 3.2.1.86), amino acid metabolism (e.g., EC 1.1.1.100, EC 1.6.5.3), and nucleotide metabolism (e.g., EC 2.7.7.7, EC 2.7.7.6), exhibited comparable abundance across groups.

Although some minor variations were observed in individual enzymes, the differences did not form consistent patterns related to host health or pathogenicity. Importantly, no significant enrichment of enzymes related to lipopolysaccharide biosynthesis, antibiotic resistance, or virulence was detected.

These results indicate that oral administration of MNN did not substantially alter the functional capacity of the gut microbiota, thereby further supporting its in vivo safety.

### 3.8 Genomic features

**Figure 6:**
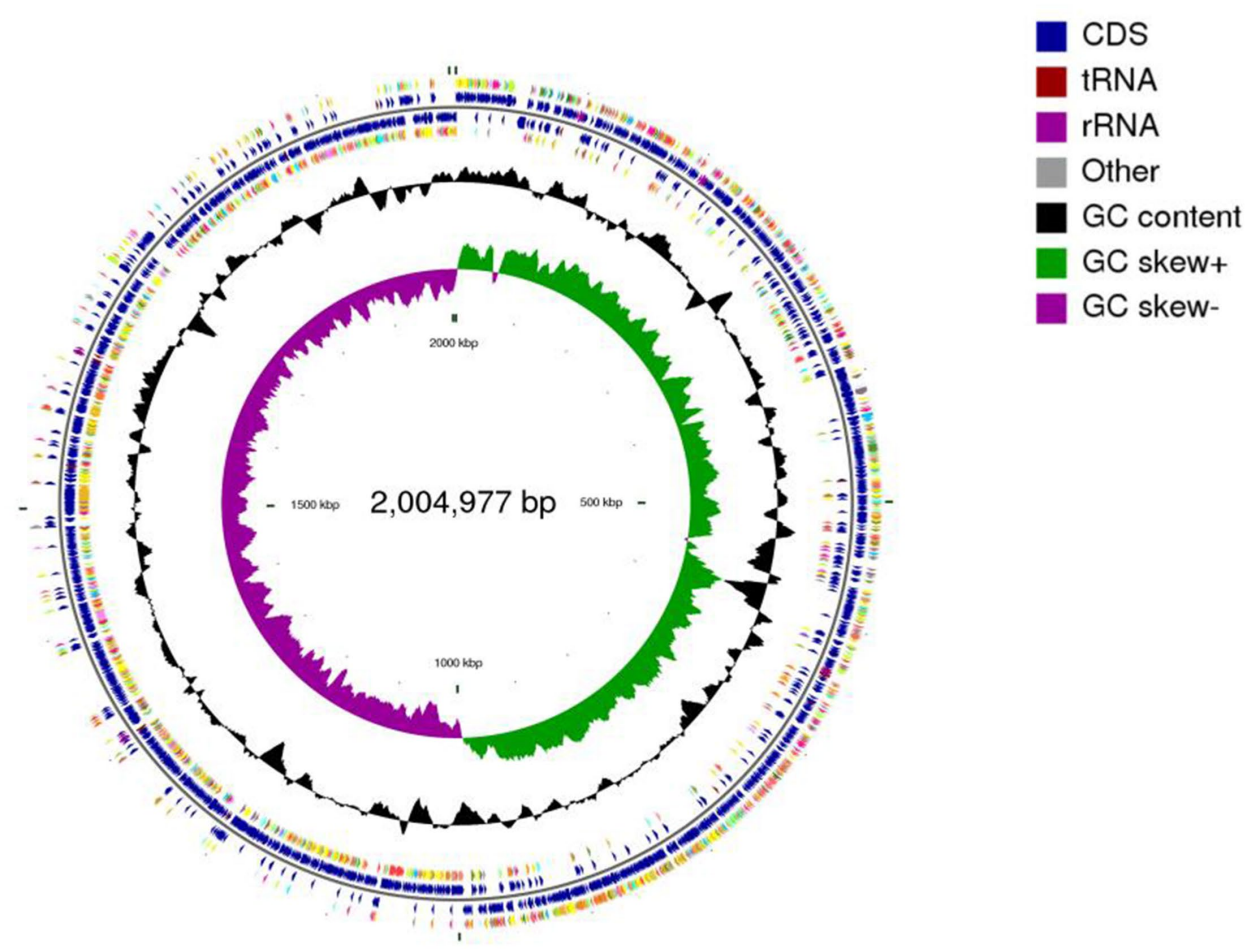
Complete genome map of *L. plantarum* MNN. Circles are indicated from outside to inside: circles 1 and 2 (blue) indicate forward and reverse strands, which represent genes for CDS, tRNA, and rRNA. Circle 3 (black) indicates the GC percentage of the genome. Circle 4 (purple and green) represents GC skew.

**Figure 7:**
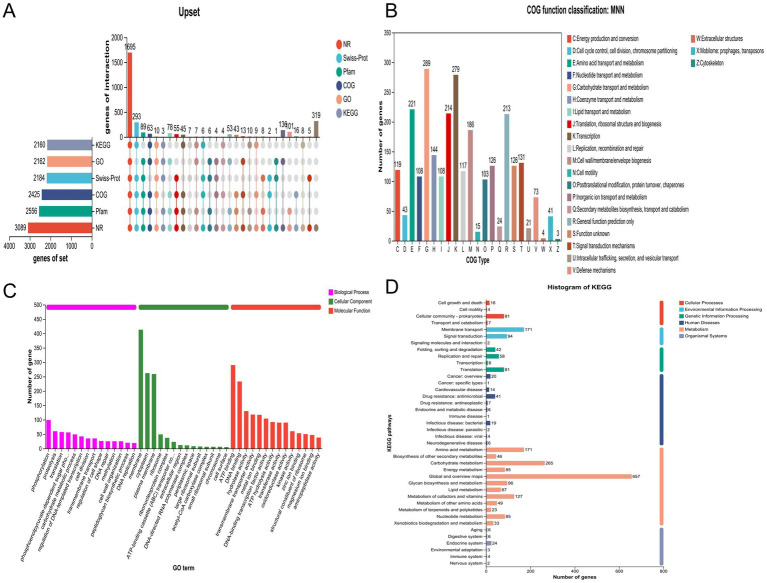
Prediction proteins function of *L. plantarum* MNN. **(A)** General database annotation percentage statistics; **(B)** COG of proteins functional; **(C)** GO analysis; and **(D)** KEGG pathways enrichment.

Whole-genome sequencing revealed a single circular chromosome in *L. plantarum* MNN, measuring 3,297,815 bp. The genome had a G + C content of 44.53% (Figure 6). The circular genome map with annotated coding sequences (CDSs) is shown in Figure 7A. Functional annotation based on the COG database classified 2,425 genes into different categories (Figure 7B). Gene Ontology (GO) enrichment analysis is shown in Figure 7C. KEGG annotation assigned 2,434 genes to 40 functional pathways. The most enriched categories were overview maps, carbohydrate metabolism, and membrane transport. These categories included 657, 265, and 171 genes, respectively (Figure 7D). Annotation further predicted 3,091 coding sequences (CDSs) with a total length of 2,773,341 bp (Table 2). In addition, 199 pseudogenes, 16 rRNA genes, 67 tRNA genes, and 43 sRNA genes were identified (Table 3). Additional genomic features included eight genomic islands, three prophage regions, 70 repeat sequences totaling 34,967 bp, one insertion sequence, and 292 putative virulence-related. A total of 112 carbohydrate-active enzymes (CAZymes) were identified (Table 4). These included 52 glycoside hydrolases (GHs) and 34 glycosyltransferases (GTs).

**Table 3:** General genomic information of the strain MNN.

Indicator	Number or content
Chromosome (bp)	3,297,815
G + C content of chromosome (%)	44.53
Coding gene numbers	3,091
Total length of coding genes (bp)	2,773,341
Pesudogene size (bp)	235,610
Pesudogene number	199
rRNAs (16 S–23 S-5S)	16
tRNA	67
sRNA	43

**Table 4:** CAZymes-encoding genes of *Lactobacillus johnsonii* MNN.

CAZymes class definition	Gene counts
Auxiliary Activities	11
Carbohydrate Esterases	15
Glycoside Hydrolases	52
Glycosyl Transferases	34

**Figure 8:**
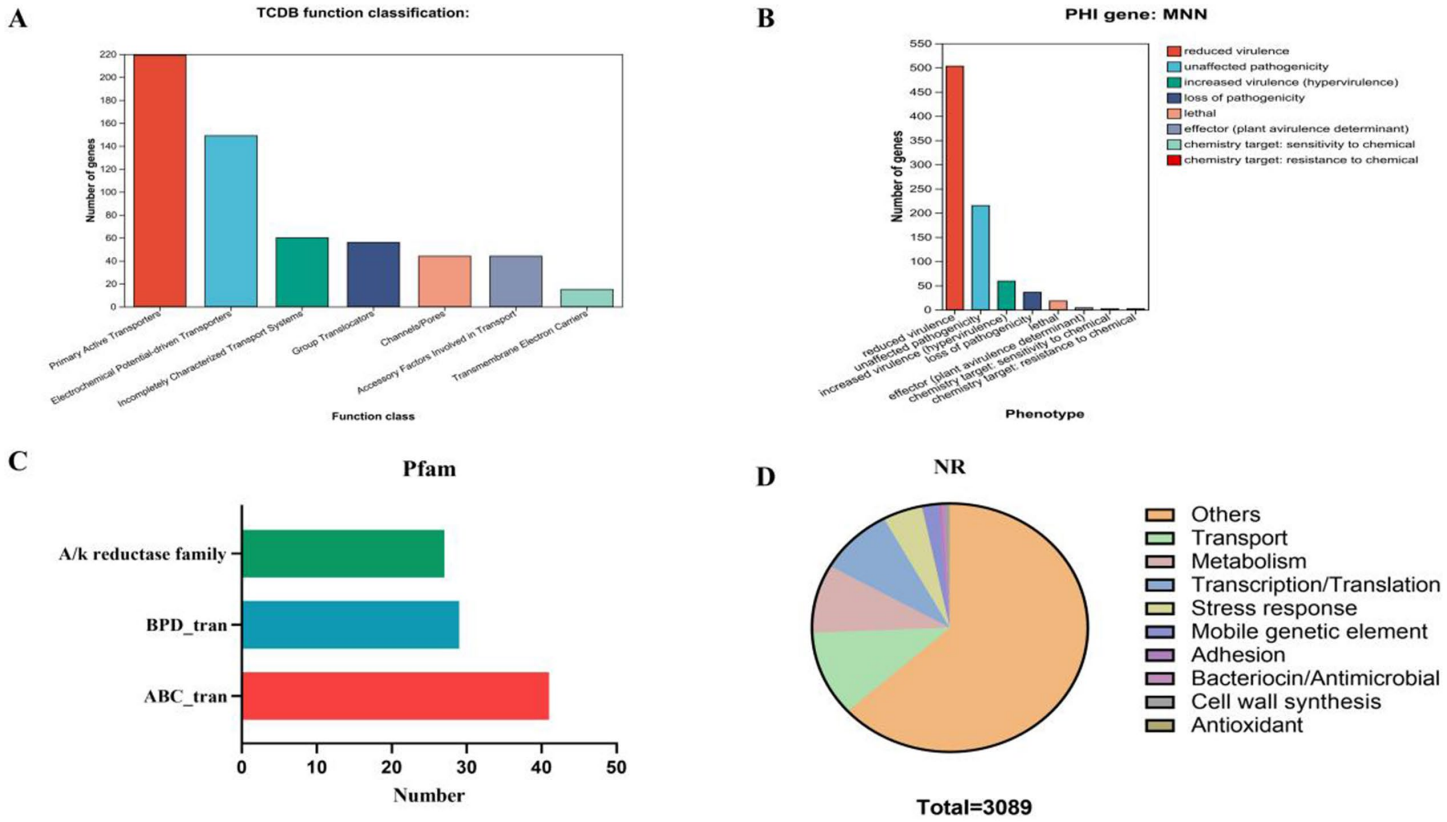
Proprietary database annotations of *L. plantarum* MNN: **(A)** Pathogen host interactions annotations; **(B)** TCDB transporter protein; **(C)** PFAM domain; and **(D)** NR annotations of MNN.

**Figure 9:**
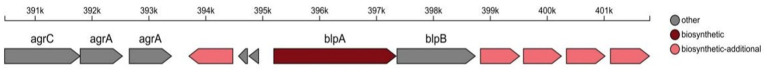
Region of RiPP-like of MNN.

Further annotation identified 587 membrane transport proteins and 719 pathogen–host interaction (PHI) genes (Figure 8A), with primary active transporters representing the largest group (219 proteins) (Figure 8B). Domain analysis identified ABC transporters (ABC_tran, 41 counts), binding-protein-dependent transport system inner membrane components (BPD_tran, 29 counts), and aldo/keto reductase family proteins (27 counts) as the most prevalent domains (Figure 8C). Protein classification based on the non-redundant (NR) database yielded results consistent with 16S rRNA sequencing (Figure 8D). CRISPR prediction indicated the presence of one CRISPR array (MinCED, v0.4.2). Moreover, four nonribosomal secondary metabolite biosynthetic gene clusters were identified, including a RiPP-like cluster, a type III polyketide synthase (T3PKS) cluster, a terpene biosynthetic cluster, and a cyclic lactone autoinducer cluster (Figure 9).

## 4 Discussion

Breast milk represents a natural reservoir of beneficial microorganisms that play pivotal roles in establishing neonatal gut microbiota and supporting immune maturation (Łubiech and Twarużek, 2020; Fagan et al., 2024). While lactic acid bacteria (LAB) isolated from human and bovine milk have been extensively studied, feline milk remains an underexplored microbial niche (Pellegrino et al., 2019; Navarré et al., 2024). The present study isolated and characterized a novel strain, *L. plantarum* MNN, from feline milk and comprehensively assessed its probiotic potential through genomic, *in vitro*, and *in vivo* analyses. Unlike previous studies focusing primarily on safety and phenotypic properties, this research integrates host-adapted functional genomics and gut microbiota profiling, providing mechanistic insights into how a feline milk-derived LAB may interact with the gastrointestinal ecosystem and maintain microbial homeostasis.

The ability of probiotic strains to withstand the harsh gastrointestinal environment is fundamental to their efficacy. The selection of a 3 h exposure period was intended to reflect physiologically relevant gastrointestinal transit rather than prolonged stress conditions. While longer exposure times may provide additional information on extreme tolerance, the present design focuses on conditions most relevant to in vivo passage. *L. plantarum* MNN demonstrated exceptional tolerance to acidic and bile conditions, with survival rates of 99.83% at pH 2.5 and 88% under 0.3% bile salt stress, outperforming many previously reported *L. plantarum* isolates. Genome annotation identified several stress response genes—including groEL, dnaK, clpB, and trxA—that encode molecular chaperones and redox regulators associated with acid and bile resistance. The presence of a cation-transporting P-type ATPase and bile salt hydrolase (BSH) genes further explains the observed physiological resilience. These genetic features suggest that MNN possesses an inherent adaptive advantage in the feline intestinal tract, which is characterized by rapid transit time and high bile acid flux due to the carnivorous diet of cats. Hence, MNN represents a host-adapted LAB capable of surviving the unique gastrointestinal environment of companion animals.

Oxidative stress contributes to intestinal inflammation and epithelial damage in both humans and animals (Melnikov et al., 2002; Amaretti et al., 2013). The strong antioxidant activities of MNN, reflected by DPPH, ABTS, and superoxide scavenging capacities, were corroborated by in vivo findings showing increased serum glutathione (GSH) and superoxide dismutase (SOD) levels alongside decreased malondialdehyde (MDA) concentrations (Amaretti et al., 2013; Zhou et al., 2023). Genomic annotation revealed multiple genes related to oxidative defense, including katA (catalase), gshA (glutathione biosynthesis), which collectively form an antioxidant network that mitigates reactive oxygen species (ROS). Notably, pyridoxamine 5′-phosphate oxidase and NADH-dependent nitroreductase, identified in the genome, may enhance redox cycling and protect the bacterial cells and host tissue from oxidative damage. These results suggest that MNN not only resists oxidative stress but may also contribute to reinforcing the host’s antioxidative defense system—a functional trait of significant relevance in mitigating intestinal oxidative injury in pets exposed to diet or pathogen-induced stress.

LAB exert their probiotic benefits partly through the production of antimicrobial substances, including organic acids, hydrogen peroxide, and bacteriocins (Ducarmon et al., 2019; Li et al., 2022; Yaacob et al., 2022). The pronounced inhibitory effects of MNN against *E. coli* O157, *Staphylococcus aureus*, and *S. typhimurium* SL1344 indicate that both its cellular components and metabolic products possess antimicrobial activity. AntiSMASH analysis revealed a complete operon encoding plantaricin EF, a two-peptide class IIb bacteriocin system (plnE and plnF) commonly associated with *L. plantarum* (Allison et al., 1994). These peptides disrupt bacterial membrane integrity, leading to pathogen suppression. Additionally, the detection of genes involved in lactic acid and acetic acid biosynthesis supports the production of organic acids contributing to pH-mediated pathogen inhibition. Together, these findings provide genomic evidence explaining the strong antimicrobial phenotype of MNN and its potential application in controlling gastrointestinal pathogens in pets, reducing antibiotic dependence, and supporting the “One Health” antimicrobial stewardship initiative. Bacteriocin and organic acid production were not directly quantified in this study. Antimicrobial activity was evaluated phenotypically, and genomic analysis suggested the presence of related biosynthetic genes.

Effective adhesion to intestinal epithelial cells facilitates colonization, competitive exclusion of pathogens, and modulation of mucosal immunity (Marín et al., 1997; Poimenidou et al., 2023). MNN exhibited high surface hydrophobicity (51.48%) and notable adhesion to Caco-2 cells (31.9%). Genome analysis revealed multiple adhesion-related factors, including elongation factor Tu (EF-Tu), several LPXTG-anchored surface proteins and MucBP/mucin-binding domain-containing proteins, which are known to mediate mucus interaction and host–microbe adhesion in lactic acid bacteria. These features may promote persistence in the gastrointestinal tract and stable colonization of the host epithelium. Furthermore, several transporter systems—including ABC-type amino acid and sugar transporters—were identified, suggesting that MNN efficiently acquires nutrients in the gut environment, further enhancing its ecological competitiveness.

Unlike most previous probiotic studies that focus solely on host biochemical markers, this work incorporated 16S rRNA sequencing to evaluate the influence of MNN on the gut microbiota composition (Derrien and van Hylckama Vlieg, 2015). Interestingly, oral administration of MNN did not significantly alter *α*- or *β*-diversity indices, indicating that MNN preserves microbial stability rather than perturbing community structure. Although the ACE index exhibited a decreasing trend in the MNN-treated group, the difference was not statistically significant. Given the inherent variability of alpha diversity metrics, subtle changes in microbial richness may require larger sample sizes to be robustly detected, and thus this trend should be interpreted with caution. This is a desirable characteristic, as excessive microbiota modulation may induce dysbiosis in healthy hosts. Gut microbiota composition analysis revealed that MNN administration induced a moderate but biologically meaningful reshaping of the intestinal microbial community at both the phylum and genus levels. At the phylum level, *Bacillota* and *Bacteroidota* remained the dominant taxa in both groups, consistent with the typical murine gut microbial structure. Notably, MNN supplementation increased the relative abundance of Bacillota while slightly reducing *Bacteroidota*, suggesting a shift toward a microbial configuration commonly associated with improved intestinal barrier integrity and metabolic homeostasis. Such modulation of the *Bacillota/Bacteroidota* balance has been frequently linked to enhanced epithelial function and host immune regulation. At the genus level, MNN treatment was characterized by a marked enrichment of *Ligilactobacillus* and *Limosilactobacillus*, indicating successful intestinal colonization or stimulation of beneficial lactic acid bacteria. These genera are well known for their roles in maintaining gut barrier function, producing antimicrobial metabolites, and competitively excluding opportunistic pathogens. In contrast, the relative abundance of genera such as *Desulfovibrio* and *Mucispirillum*, which have been associated with mucosal irritation, hydrogen sulfide production, and inflammation under dysbiotic conditions, was reduced in the MNN group compared with the control group. This shift suggests that MNN may indirectly suppress potentially harmful taxa through niche competition or metabolite-mediated mechanisms. Genome annotation identified 292 genes annotated as putative virulence-related factors based on sequence homology. It should be emphasized that such annotations are largely predictive and do not necessarily indicate functional virulence. Many of these genes are commonly found in non-pathogenic lactic acid bacteria and are often involved in basic cellular processes, stress responses, adhesion, or host interaction rather than toxin production or invasive behavior. Importantly, no complete virulence operons, known toxin genes, or secretion systems typically associated with pathogenicity were identified in the MNN genome. Similar observations have been reported in other *Lactiplantibacillus* and *Pediococcus* strains that are widely regarded as safe and have been used as probiotics. Therefore, the presence of putative virulence-related genes should be interpreted with caution and in the context of their genomic organization and functional relevance. When combined with the absence of hemolytic activity, the lack of antibiotic resistance concerns, and the favorable *in vivo* safety outcomes observed in murine models, these genomic findings collectively support the biosafety of strain MNN for further probiotic development. The identification of a CRISPR array in the genome of strain MNN suggests a potential role in defense against bacteriophage infection and maintenance of genomic stability. CRISPR–Cas systems are widely recognized as adaptive immune mechanisms in bacteria and have been reported in many lactic acid bacteria, where they may contribute to protection against horizontal gene transfer and phage predation. However, it should be noted that the presence of a CRISPR array alone does not confirm its functional activity. In the present study, no experimental assays were conducted to verify CRISPR-mediated interference or spacer activity. Therefore, conclusions regarding phage resistance or functional immunity should be interpreted cautiously. From a biosafety perspective, the presence of a CRISPR array may nonetheless be considered a favorable genomic feature, as it could contribute to genome stability and reduce the likelihood of acquiring undesirable genes through horizontal gene transfer. Further studies are required to experimentally validate the activity and functional relevance of the CRISPR system in strain MNN. In the present study, preservation of gut microbial homeostasis refers to the maintenance of overall community structure and the absence of broad dysbiosis, rather than the complete lack of compositional changes. LEfSe analysis revealed minor enrichment of genera such as Oscillibacter and Adlercreutzia—both associated with anti-inflammatory and butyrate-producing capacities—suggesting that MNN may subtly promote beneficial taxa without disrupting microbial equilibrium (Markowiak and Śliżewska, 2017). While LEfSe analysis identified several differentially abundant taxa following MNN administration, these alterations were moderate and limited to specific bacterial groups, without disrupting the dominant phyla or overall microbial architecture. Such targeted shifts in microbial composition are commonly observed following probiotic intervention and are generally considered indicative of adaptive modulation rather than pathological imbalance. Importantly, the taxa enriched in the MNN-treated group have been frequently associated with beneficial metabolic functions and intestinal health, supporting the notion that MNN promotes a stable yet functionally optimized gut microbial ecosystem rather than inducing dysbiosis. Functional prediction based on PICRUSt2 was employed in this study to infer potential metabolic shifts in the gut microbiota following MNN administration. PICRUSt2 enables the estimation of functional profiles from 16S rRNA gene data by leveraging reference genomes and phylogenetic relationships, and has been widely used as a hypothesis-generating tool in microbiota studies. The predicted enrichment of pathways related to carbohydrate metabolism and microbial homeostasis is consistent with the observed compositional changes at the taxonomic level. Nevertheless, it should be acknowledged that PICRUSt2 predictions are inherently limited by their reliance on 16S rRNA gene sequences and reference genome databases, and therefore cannot fully capture strain-level functional variation or actual gene expression. As such, the predicted enzymatic functions should be interpreted as indicative rather than definitive. Metagenomic or metatranscriptomic sequencing would provide higher functional resolution and enable direct validation of these predicted pathways. Although such approaches were beyond the scope of the present study, they represent important directions for future research to further elucidate the functional mechanisms underlying the probiotic effects of strain MNN. Functional prediction via PICRUSt2 showed that metabolic pathways related to carbohydrate and amino acid metabolism remained stable, and no enrichment of antibiotic resistance or virulence-associated enzymes was detected. These results collectively demonstrate that MNN maintains gut ecological homeostasis, representing a safe and balanced probiotic intervention for companion animals.

Although strain MNN was isolated from feline milk, its functional evaluation in the present study was conducted exclusively using murine models. Mice are widely used as a well-established and reproducible system for initial probiotic safety and efficacy assessment; however, species-specific differences in gut physiology, immune responses, and microbiota composition should be acknowledged. Therefore, conclusions regarding host-specific adaptation of strain MNN are primarily inferred from its feline milk origin and genomic characteristics rather than direct functional validation in feline hosts. The identification of genes associated with stress tolerance, adhesion, and metabolic adaptability suggests that MNN possesses traits potentially favorable for survival and function in the gastrointestinal environment. Nevertheless, extrapolation of murine results to feline applications should be made with caution. Future studies employing feline-specific *in vitro* systems or *in vivo* models will be necessary to further validate host-adapted probiotic effects and confirm translational relevance in companion animals.

It should be noted that *Pediococcus acidilactici* M22 was not included as an experimental control in the present study. Comparisons with M22 were made based on previously published data and are intended to provide contextual reference rather than direct experimental comparison. Future studies incorporating head-to-head comparisons will be valuable to further delineate strain-specific differences. Compared with our previously reported *P. acidilactici* M22 strain from feline milk, *L. plantarum* MNN exhibits a larger genome size (3.29 Mb vs. 2.06 Mb) and a richer gene repertoire related to membrane transport, redox regulation, and carbohydrate metabolism. This expanded genomic versatility may account for its superior tolerance and antioxidant activity. Importantly, while M22 primarily improved systemic antioxidant capacity, MNN additionally demonstrated the ability to preserve gut microbiota balance, representing a functional progression from safety assessment to ecological validation. Thus, MNN may serve as a next-generation host-adapted probiotic with dual antioxidant and microbiota-stabilizing capabilities.

Future research should focus on elucidating the molecular signaling pathways by which MNN interacts with host cells, such as NF-κB or Nrf2-mediated antioxidant responses, and on verifying its immunomodulatory potential through cytokine profiling (e.g., IL-6, IL-10, TNF-*α*, sIgA). Moreover, metabolomic analyses of short-chain fatty acids (SCFAs) could clarify its contributions to gut barrier integrity and metabolic health. The potential synergy between MNN and prebiotics such as galacto-oligosaccharides (GOS) or xylo-oligosaccharides (XOS) also warrants exploration, as such synbiotic formulations may further enhance its colonization and bioactivity in pet gut environments.

## 5 Conclusion

In summary, *L. plantarum* MNN isolated from feline milk demonstrates a rare combination of acid and bile resistance, strong antioxidant and antimicrobial activities, and the capacity to maintain gut microbial equilibrium. Integrating genomic, functional, and ecological evidence, this study advances our understanding of host-derived probiotics by proposing a mechanistic model in which MNN promotes gut homeostasis through stress adaptation, antioxidative defense, and balanced microbial modulation. These findings not only highlight MNN as a safe and functionally robust probiotic candidate for pet nutrition but also provide a foundation for future development of precision, host-specific probiotic formulations for companion animals.

## Data Availability

The raw sequence data have been deposited in the NCBI database under accession number PRJNA1285844.
